# Symptoms of depression and anxiety in Chinese adolescents: heterogeneity and associations with executive function

**DOI:** 10.1186/s12888-023-04810-z

**Published:** 2023-06-07

**Authors:** Jing Sun, Shaoxia Wang, Guoxia Mu, Jingru Liu, Rina Su, Xiang Zhang, Jianqun Fang, Yanrong Wang

**Affiliations:** 1 School of Nrising, Ningxia Medical University, No. 1106 South Shengli Street, Yinchuan, 750004 Ningxia China; 2 Mental Health Center, General Hospital of Ningxia Medical University, No. 804 South Shengli Street, Yinchuan, 750004 Ningxia China

**Keywords:** Depression, Anxiety, Latent profile analysis, Executive function

## Abstract

**Background:**

Depression and anxiety are common symptoms associated with significant morbidity in adolescents. Few studies have explored the relationship between latent profiles of adolescent depression-anxiety symptoms and executive function (EF), which is also a major pediatric public health concern.

**Methods:**

The sample included 1,306 participants who were recruited from two schools in Ningxia. The Depression Self-Rating Scale for Children (DSRSC) and the Screen for Child Anxiety Related Emotional Disorders (SCARED) were used to assess the level of depression-anxiety symptoms in adolescents, and their executive function state was assessed using the Behavior Rating Inventory of Executive Function-Self-Report version (BRIEF-SR). Latent profile analysis (LPA) was carried out using Mplus 7.0 to explore the most likely number of profiles based on the subscales of DSRSC and SCARED. The relationship between adolescents’ executive function and depression-anxiety symptoms were analyzed by multivariable logistic regression, and the odds ratio were used to test the impact of this relationship.

**Results:**

The LPA results show that the three-profile model was the best-fitting model for adolescent depression and anxiety symptoms. The proportions of Profile-1 (“Healthy Group”), Profile-2 (“Anxiety Disorder Group”), and Profile-3 (“Depression-Anxiety Disorder Group”) were 61.4%, 23.9%, and 14.7%, respectively. Additional analyses using multivariable logistic regression suggested that poor shifting capacity and emotional control were significantly more likely to be classified into the depression and/or anxiety groups, and worse working memory, task completion, and better inhibition were significantly more likely to be classified into the anxiety group.

**Conclusions:**

The findings contribute to our understanding of the heterogeneity of adolescents’ depression-anxiety symptoms and highlight the important role of executive function in influencing mental health outcomes. These findings will guide the improvement and delivery of interventions for the treatment of anxiety and depression in adolescents, mitigating functional impairments in patients and reducing disease risk.

## Background

Depression and anxiety are the most prevalent and onerous mental health problems among adolescents worldwide [1] and are profoundly concerning. Research on the global prevalence of mental disorders among children and adolescents indicate that approximately 25% of adolescents suffer from depression and anxiety [2, 3]. According to representative epidemiological studies conducted nationwide, the prevalence of mental health problems among individuals aged ≤ 18 years in China in the past year increased from 11.46% in 2008/2009 to 18.1% in 2019/2020 [4]. Empirical studies indicate that mental health problems are significant risk factors for poor social-psychological outcomes and behavioral problems; these problems also intensify and complicate several illnesses among adolescents [5–7]. The high rates of mental health problems among adolescents and substantial costs of their treatment necessitate the identification of risk factors for depression and anxiety among adolescents.

The role played by executive function (EF) is an important factor in revealing the complex constellations of biological and environmental effects on mental health problems. EF refers to high-order cognitive processing that allows for intentional and goal-directed problem-solving and adaptation [8]. It is a multi-faceted construct involving inhibitory control, task-shifting, emotional control, monitoring, working memory, planning and organization among other skills. Certain empirical studies show that executive dysfunction underlies most mental disorders [9], and poor EF can negatively impact emotional regulation, which may, in turn, lead to future depression and anxiety [10]. Previous studies on the relationship between EF and anxiety and depression among the youth have mainly focused on the association of EF with a single mental health problem. Gillespie et al. [11] explored the relationship between depression and EF and found that disordered EF for adolescents was associated with severe depression. Barbara et al. [12] highlights that EF is significantly related to anxiety in children and adolescents. Although these studies have provided substantial insight into the contribution of EF to internalizing symptoms, past studies present various mental health problems as independent entities. These studies fail to consider the high rates of observed comorbidity. However, epidemiological studies have demonstrated that comorbidity is the rule rather than the exception; therefore, solely exploring the association of EF with a single mental health problem may not be comprehensive [13]. Determining the association of depression and anxiety with EF in adolescents is essential to elucidating internalizing disorders as well as more optimized interventions for anxiety and depression among the youth.

Numerous studies on mental health problems suggest common risk factors and high rates of comorbidity for depression and anxiety [14–16]. Previous studies have confirmed heterogeneity among individuals with depression and anxiety symptoms [17]. For instance, two people with similar diagnoses may exhibit different symptoms; certain individuals exhibit high depression and low anxiety levels, while others exhibit low depression and high anxiety levels [18]. Previous studies have mainly used variable-centered approaches, which are based on the assumption of homogeneity within a sample, without considering individual factors and disregarding important information on individuals. This is not conducive to elucidating the heterogeneity of depression and anxiety symptoms. The heterogeneity or group-specific distribution of symptoms, particularly natural clustering, has been relatively disregarded in the assessment of mental health [19]. To address this limitation and elucidate mental health problems, recent research has used latent profile analysis (LPA) to explore the patterns of depression and anxiety. LPA focuses on the heterogeneity of a population, and individuals with similar characteristics are grouped together according to distinct subtypes based on their response patterns [20]. This facilitates the tailoring of prevention interventions to the varied needs of different subgroups for enhancing their psychological health. Although studies have investigated the heterogeneity of depression and anxiety using a person-centered approach [18, 21–24], they had small sample sizes or used research tools that were cumbersome. Therefore, expanding the sample size and employing easy-to-use tools are essential to identifying potential patterns of mental health problems. In addition, most studies only focus on the association between EF and depression or anxiety, disregarding the association between EF and the heterogeneity of mental health problems. This may conceal significant information on the individual and hinder the provision of specific reference for future individualized intervention research on different types of depression and anxiety; this challenge can be overcome by employing LPA.

Using LPA, we study common patterns of depression and anxiety in adolescents and identify specific aspects of EF possibly associated with depression and anxiety subtypes. Similar to previous studies, we hypothesize that mental health problems among the youth in China have latent profiles (e.g., low depression and anxiety, low depression and high anxiety, and high depression and anxiety) [22]. Moreover, people classified into different depression and anxiety subgroups were associated with different dimensions of EF [25, 26]. By studying the relationship between the heterogeneity of depression and anxiety symptoms and EF, we hope to identify specific subgroups that might be most at risk. By matching tailored programs to different subgroups and targeting high-risk adolescents, resources can be leveraged more effectively for the prevention and treatment of mental health problems.

## Methods

### Participants and procedures

We invited a middle school and an elementary school in Yinchuan to participate in this cross-sectional study. The Chinese government has made great effort to create an equitable educational environment, despite the disparity in students’ innate abilities. The schooling process should provide the fairest possible opportunity for each child, where students are assigned to the school closest to where their families reside, thus preventing cases of unequal allocation of educational resources. Therefore, the students from these two schools can be representative of the average Chinese youth. Students were selected via a stratified cluster group sampling method and two to three classes were randomly selected for each grade. The final sample comprised 1,306 adolescents from two schools. The original data were collected from 1,332 participants; however, the sample was pared down (i.e., *N* = 1,306) after eliminating questionnaires with non-valid responses (e.g., missing key information on depression, anxiety, or executive function). Participants’ ages ranged from 10–18 years (*M ± SD* = 13.98 ± 1.19). The sample comprised 697 girls (53.4%) and 609 (46.6%) boys. Most participants had at least one sibling (61.5%) and were from the Han ethnic group (90.12%). Most participants ranked in the bottom five of their class (40.3%). Furthermore, 9% of the participants reported drinking alcohol and 3.8% reported smoking.

The study procedure was approved by the first author’s institutional review board. After obtaining the cooperation of school administrators and the informed consent of parents and students, the survey was conducted in classrooms by research staff. After the instructions were explained, the participants independently completed all questions in the questionnaire. It took approximately 15–20 min to complete the questionnaire, and all questionnaires were distributed and collected in one sitting.

### Measures

#### Depression self-rating scale for children

The severity of depression was assessed using the Depression Self-Rating Scale for Children (DSRSC). The self-report instrument has been modified and validated for use among Chinese-speaking populations [27]. The scale contains 18 items evaluated on a 3-point Likert-scale (0 = “*None*,” 1 = “*Sometimes*,” 2 = “*Always*”). The total score ranges from 0 to 36, and a score of ≥ 15 is indicative of depression. Higher scores indicate relatively severe depression. The internal consistency (Cronbach’s alpha) of the DSRSC in this study was 0.68.

#### Screen for child anxiety related emotional disorders

We assessed participants’ anxiety symptoms using the Screen for Child Anxiety Related Emotional Disorders (SCARED) [28], which constitutes a 41-item questionnaire evaluated on a 3-point Likert scale (0 = “*None*,” 1 = “*Sometimes*,” 2 = “*Always*”), and comprises five subscales: somatic/panic symptoms (13 items), generalized anxiety (9 items), separation anxiety (8 items), social phobia (7 items), and school phobia (4 items). The total score can range from 0 to 82, with scores above 23 denoting significant anxiety. Internal consistency (Cronbach’s alpha) in this study was 0.97.

#### Behavior rating inventory of executive function-self-report version

The Behavior Rating Inventory of Executive Function-Self-Report Version (BRIEF-SR) was used to assess EF. All 80 items are rated on a 3-point Likert scale (1 = “*Never*,” 2 = “*Sometimes*,” and 3 = “*Often*”), and comprise eight subscales: inhibitory control, emotion shift capacity, emotional control, monitoring, working memory, planning/organization, material organization, and task completion. *T* scores were used to interpret the adolescents’ self-reported level of EF on the BRIEF-SR rating form. These scores are linear transformations of the scores from the raw scale (*M* = 50, *SD* = 10). Traditionally, *T* scores of ≥ 65 are considered clinically significant; however, in the case of the BRIEF-SR, *T* scores ranging between 60 and 64 on any of the clinical scales or indices may warrant clinical interpretation. Higher scores typically indicate relatively poor performance. Internal consistency (Cronbach’s alpha) of the BRIEF-SR in this study was 0.982.

### Data analysis

LPA was conducted using Mplus 7.0 to explore the most likely number of profiles based on the DSRSC and subscales of SCARED. The score of each subscale was standardized using a *Z*-score for statistical analysis [29]. The Akaike Information Criterion (AIC), Bayesian Information Criterion (BIC), and adjusted Bayesian Information Criterion (aBIC) are commonly used to compare different competing models, with the lowest value on each criterion indicating the best-fitting model [30]. Considering the Lo-Mendell-Rubin (LMR) test, a low and significant *p*-value signifies that the estimated model is superior to the model with one profile less [31]. Entropy can further be used to evaluate the quality of each profile solution resulting from LPA, and values exceeding .80 are preferred.

After identifying the appropriate number of latent profiles, the distribution of and associations with demographic characteristics according to profiles were examined using a series of cross-tabulations and bivariate analyses (using Chi-square tests and Analysis of variance [ANOVA]). Finally, SPSS 25.0 was used for multinomial logistical regression to validate the association between depression and anxiety profiles and several aspects of EF, with three latent profile solutions serving as the dependent variable. A *p* value < 0.05 was considered statistically significant (double-sided).

## Results

### Latent profiles of depression and anxiety symptoms in adolescents

Fit indicators for the different LPA models are presented in Table 1. Among the models examined, a three-profile solution demonstrated the lowest AIC, BIC, and aBIC values as well as the highest entropy. Thus, a three-profile pattern of depression and anxiety symptoms in adolescents was the best-fitting model.

**Table 1:** Model fit indices for one- to six-profile patterns of depression and anxiety in adolescents and profile prevalence (%) from LPA (*N* = 1306)

No. of profiles	AIC	BIC	aBIC	LMR	Entropy	Profile prevalence					
						1	2	3	4	5	6
1	22255.61	22317.70	22279.58								
2	17976.08	18074.40	18014.05	< 0.001	0.96	0.780	0.220				
3	16944.03	17078.57	16995.98	< 0.001	0.91	0.614	0.239	0.147			
4	16180.73	16351.49	16246.67	> 0.05	0.92	0.229	0.579	0.151	0.041		
5	15947.26	16154.25	16027.19	< 0.05	0.90	0.534	0.226	0.098	0.106	0.036	
6	15736.56	15979.77	15830.47	> 0.05	0.91	0.014	0.089	0.223	0.535	0.103	0.036

Note: The values in the LMR columns are the *p* values related to LMR in comparing fit between models. AIC = Akaike Information Criterion; BIC = Bayesian Information Criterion; aBIC = adjusted Bayesian Information Criterion; LMR = Lo-Mendell-Rubin; LPA = Latent Profile Analysis

**Fig. 1:**
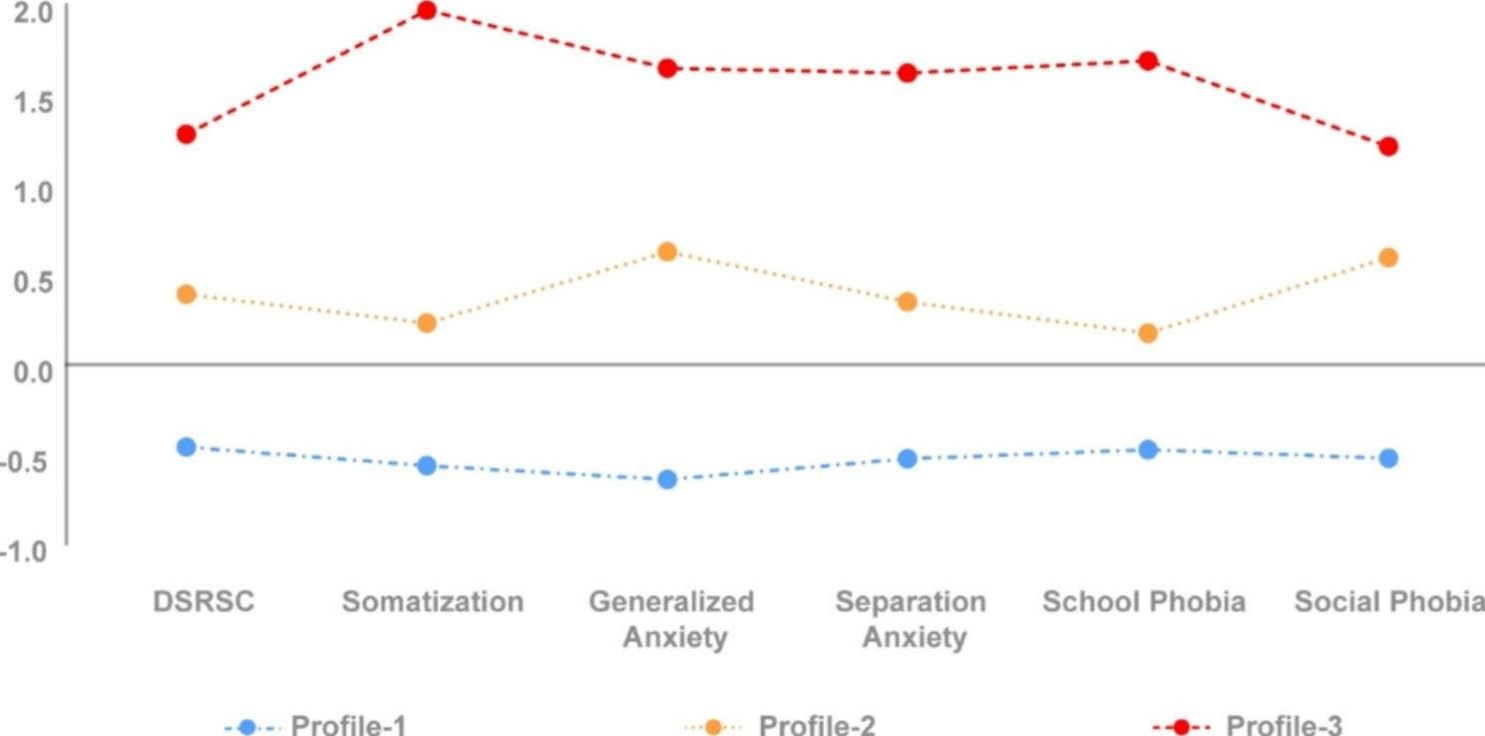
Depression-anxiety profiles (*Z* scores)

The three-profile solution (as illustrated in Fig. 1) was retained for further interpretation. Table 2 displays the means and standard deviations of the DSRSC total score and SCARED subscales for each of the three profiles. We calculated the correlation between the DSRSC and SCARED scale sum scores using Pearson’s method (*r* = 0.627, *p* < 0.001). The first and largest profile represented 61.6% (n = 805) of the respondents and was characterized by the lowest self-reported DSRSC and SCARED scores. This subgroup of adolescents self-reported the fewest symptoms of depression and anxiety and was labelled as the healthy group. The second profile represented 23.7% (n = 309) of the respondents, who scored above-average SCARED levels but scored a DSRSC mean below clinical cutoff, suggesting that this profile constituted adolescents with self-reported anxiety symptoms without clinical depression. This profile was labelled the anxiety symptom group. The third profile represented 14.7% (n = 192) of the respondents, whose scores reflected the most severe self-reported symptoms of depression and anxiety. This profile was labelled the depression-anxiety symptom group.

**Table 2:** Mean and standard deviation for DSRSC total score and SCARED subscales of the three profiles of depression-anxiety (*N* = 1306)

Variable	Profile-1 n = 805		Profile-2 n = 309		Profile-3 n = 192	
	Mean	SD	Mean	SD	Mean	SD
DSRSC	6.91	5.36	13.00	5.78	19.33	5.56
Somatization	1.01	1.41	5.42	3.00	14.87	4.63
Generalized Anxiety	1.40	1.64	6.88	2.40	11.18	3.16
Separation Anxiety	1.09	1.37	3.70	2.13	7.37	2.88
School Phobia	0.29	0.66	1.39	1.33	3.91	1.78
Social Phobia	2.06	2.15	6.21	3.19	8.37	3.32
SCARED	5.87	4.83	23.61	6.00	45.72	10.75

Note: DSRSC = Depression Self-Rating Scale for Children; SCARED = Screen for Child Anxiety Related Emotional Disorders; Profile − 1 = healthy group; Profile − 2 = anxiety symptom group; Profile-3 = depression-anxiety symptom group

Table 3 shows the socio-demographic characteristics of the three profiles. No statistically significant differences were observed among the profiles, except for sex, study rank, and substance use (including smoking and drinking). Girls showed the highest rate of anxiety symptoms (59.9%), and adolescents without depression or anxiety symptoms had the highest study rankings (42.2%). The percentage of substance use was highest in adolescents with symptoms of depression and anxiety (12.5% for smoking and 24% for drinking).

**Table 3:** Demographic characteristics in each profile (*N* = 1306)

Variables	Profile-1 n (%)	Profile-2 n (%)	Profile-3 n (%)	* F*/*X*^*2*^	*P*
Age	13.93(1.206)	14.08(1.225)	14.06(1.084)	2.277	> 0.05
Sex				9.67	< 0.05
Boy	402(49.9%)	124(40.1%)	83(43.2%)		
Girl	403(50.1%)	185(59.9%)	109(56.8%)		
One-Child				1.480	> 0.05
One	320(39.8%)	111(35.9%)	72(37.5%)		
More than one	485(60.2%)	198(64.1%)	120(62.5%)		
Study rank				14.55	< 0.01
Last 5	340(42.2%)	125(40.5%)	61(31.8%)		
Medium	299(37.1%)	105(34%)	69(35.9%)		
Top 5	166(20.6%)	79(25.6%)	62(32.3%)		
Substance use					
Smoking	17(2.1%)	8(2.6%)	24(12.5%)	64.55	< 0.01
Drinking	33(4.1%)	38(12.3%)	46(24%)	87.19	< 0.01

Note: Profile-1 = healthy group; Profile-2 = anxiety symptom group; Profile-3 = depression-anxiety symptom group

### Multinomial logistic regression analysis

Compared with the healthy group, results from the multivariate logistic regression analyses indicated that partial EF was significantly associated with latent profiles after adjusting for social demographic characteristic (e.g., sex, study rank, whether participant smokes or drinks). In the logistic model, only inhibitory control, emotion shift, emotional control, task completion, and working memory reached a significant level (*p* < 0.05). The results showed that the predictive effects of Profile-1 (healthy group) were used as the baseline reference category. An odds ratio (*OR*) was used as the effect amount of logistic regression. The specific results are presented in Table 4.

**Table 4:** Significant multinomial logistic regression results (*N* = 1,306)

Variables	Profile-2		Profile-3	
	*β*	*OR* (95%*CI*)	*β*	*OR* (95%*CI*)
Emotion shift	0.086^**^	1.089(1.052, 1.128)	0.111^**^	1.118(1.068, 1.169)
Emotional control	0.139^**^	1.149(1.109, 1.190)	0.205^**^	1.227(1.174, 1.283)
Inhibitory control	-0.058^**^	0.944(0.907, 0.983)	-0.004	0.996(0.946, 1.048)
Working memory	0.049^*^	1.051(1.008, 1.094)	0.040	1.041(0.984, 1.101)
Task completion	0.035^*^	1.036(1.000, 1.073)	0.014	1.014(0.964, 1.066)

Note: Profile-1 (healthy group) was regarded as the reference profile; * indicates that the significance level of the regression coefficient is *p* < 0.05; ** indicates that the significance level of the regression coefficient is *p* < 0.01

The findings demonstrate that emotion shift capacity, emotional control, inhibitory control, working memory, and task completion predicted whether the adolescent would fall into the anxiety symptom group. However, only emotion shift capacity and emotional control were significantly associated with membership in the depression-anxiety symptom group. Compared with Profile-1, participants with poor emotion shift capacity (OR = 1.089, 95% CI:1.052, 1.128), emotional control (OR = 1.149, 95% CI:1.109, 1.190) ,working memory (OR = 1.051, 95% CI:1.008, 1.094), task completion (OR = 1.036, 95% CI:1.000, 1.073), and more optimized inhibitory control (OR = 0.944, 95% CI: 0.907, 0.983) were more likely to fall under Profile-2. Furthermore, participants tended to fall under Profile-3 if they had poor emotion shift capacity (OR=,1.118 95% CI:1.068, 1.169) and emotional control (OR = 1.227, 95% CI:1.174, 1.283).

## Discussion

To the best of our knowledge, this study is the first to examine the associations between the potential categories of mental health problems and EF among Chinese adolescents. The study results complement research on adolescents’ mental health problems [32–34] and have clinical implications for adolescents with high depression and anxiety levels. The EF of adolescents who are at risk of internalization problems is concerning, and routine mental health screening for adolescents should consider this aspect.

Our LPA results suggest that the current sample of adolescents is more consistent with the three latent profiles model. In addition, consistent with these unique patterns of symptom occurrence, we found that each profile was associated with a different EF aspect. Similar to the cluster-analytic studies of mental health problems in adolescents, this study confirms depression-anxiety heterogeneity in adolescents delineated by the three main profiles of mental health problem subtypes [18].

Similar to previous studies, we identified one profile of participants with significant depression and anxiety symptoms; owing to an overlap of symptoms and shared etiological factors during adolescence, depression and anxiety disorders frequently occur simultaneously [35]. The rate of depression and anxiety symptoms in this profile was significantly lower than that of a Chinese sample studied by Zhou et al. [36]. This may be because Zhou et al. [35] used a different assessment tool, and assessment of the prevalence of anxiety and depressive symptoms was conducted during the COVID-19 pandemic. External environmental factors (particularly stressful life events) are strongly associated with the onset of depression [37–39]. Compared to the study by Zhou et al. [35], participants in the present study were not affected by stressful life events such as the COVID-19 pandemic; therefore, the prevalence of depressive symptoms was low. Meanwhile, we identified a subgroup that only exhibited anxiety symptoms. In contrast with the study performed by Yarrington et al., no subgroup showing only depressive symptoms was identified in the present study [40]. This may be because of the variations in age range, geography, or socio-cultural background of participants and the different assessment instruments. Our study results may indicate that depressive symptoms in adolescents often occur in combination with anxiety symptoms and that clinicians need to consider anxiety in the diagnosis and treatment of depression.

The current study also prioritized the determination of the partial EF that is significantly associated with the latent profiles of mental health problems. Although both profiles exhibited reduced EF, emotion shift and emotional control were found to be more associated with the depression-anxiety symptom group. In contrast, inhibitory control, task completion, and working memory were only associated with the anxiety symptom group. Previous studies have shown that poor emotion shift or emotional control may undermine resilience to coping with difficulties by attenuating effective coping skills [41]. Moreover, emotion shift capacity and emotional control underlie emotion regulation ability; lower levels of these skills are associated with reduced ability to effectively regulate emotions, which, in turn, increases vulnerability to depression and anxiety [42, 43]. Working memory and task completion are also important predictors in the present study; they indicate an individuals’ ability to finish or complete tasks appropriately [44]. According to Matthew et al., impairments in working memory can attenuate the control of the attention system, which may lead to increased anxiety [45]. Wang et al. found that failed task completion may cause anxiety, which is consistent with our findings [46]. Our results showed that task completion ability was significantly impaired in the anxiety symptom group. Overall, our results verified the correlation between poor task completion and increased severity in anxiety; we hope these results will provide a novel avenue for the promotion of interventions for patients with anxiety.

Finally, after examining the three profiles’ EF, individuals with more optimized inhibitory control were found to be more prone to anxiety. A previous study suggested that adolescents with greater inhibitory control consider themselves as generally being in control, and are typically able to resist impulses and consider consequences before acting, thereby reducing their risk of mental health problems [47]. Interestingly, this is inconsistent with the results we obtained. This discrepancy may be attributed to the cognitive resource hypothesis, which suggests that the overuse of inhibitory control could contribute to cognitive resource deficits that, in turn, contribute to the risk of anxiety (trying to remain in control limits attentional resources for emotional modulation) [48]. Therefore, clinicians should be highly alert to the possibility of emotional disorders when confronting adolescents with impairments in EF. Meanwhile, a systematic and comprehensive assessment of executive functioning should be considered in the diagnosis of anxiety and depressive disorders in adolescents. In particular, for individuals with impairments in emotion shift and emotional control but with high inhibitory control, psychiatrists may need to introduce psychological counseling and emotional regulation at an early stage. In addition, intervention training for EF is crucial. This measure will help mitigate anxiety and depression symptoms as well as disease risk in adolescents.

Generally, the current study identifies three distinct symptom subgroups in the occurrence of depression and anxiety in a sample of Chinese adolescents. The subgroup exhibiting symptoms of mental health problems displayed more obvious EF damage than healthy individuals. In addition, our findings suggest the development of individualized intervention strategies to improve mental health among adolescents based on the association of different symptom patterns of depression and anxiety with specific aspects of EF. For individuals with significant symptoms of depression and anxiety, the focus can be on improving their emotion shift and emotional control capacity. Based on the improvement of the two abilities mentioned above, those who only exhibited symptoms of anxiety should integrate the promotion of working memory and task completion abilities and make appropriate adjustments to their inhibitory control skills.

Notably, there were several limitations in the current study. First, although the application of people-centered statistical methods can be viewed as a strength, our study only investigates the relationship between EF and the depression-anxiety levels of adolescents at one point in time. It does not dynamically investigate the relationship between the two variables, which can lead to false positives [49]. In addition, all scales in the current study constituted self-report measures, which may result in expectation bias. Future research should employ objective measures, such as the clinician ratings, to confirm findings.

## Data Availability

The datasets generated and/or analyzed during the current study are available from the corresponding author, YRW, upon reasonable request, but are not publicly available due to patient privacy restrictions.

## Abbreviations

EF: Executive function
LPA: Latent Profile Analysis
DSRSC: Depression Self -rating Scale for Children
SCARED: The Screen for Child Anxiety Related Emotional Disorders
BRIEF-SR: Behavior Rating Inventory of Executive Function -Self-Report Version
AIC: Akaike Information Criterion
BIC: Bayesian Information Criterion
aBIC: Adjusted Bayesian Information Criterion
LMR: Lo-Mendell-Rubin
OR: Odds Ratio
